# Early-Onset Syringomyelia: A Rare Complication of TB Meningitis

**DOI:** 10.7759/cureus.95122

**Published:** 2025-10-22

**Authors:** Aditya K Adhikarla, Robyn Terry, Murad Ghrew, Rajiv Mohanraj

**Affiliations:** 1 Internal Medicine, Manchester Royal Infirmary, Manchester, GBR; 2 Neurology, Northern Care Alliance, Manchester, GBR; 3 Respiratory Medicine, Intensive Care Unit, Northern Care Alliance, Manchester, GBR; 4 Faculty of Biology, Medicine and Health, University of Manchester, Manchester, GBR

**Keywords:** cervical syrinx, extrapulmonary tuberculosis (eptb), neurological infections, paradoxical reaction, syringomyelia, tb meningitis, tuberculous granuloma

## Abstract

Syringomyelia is a rare but recognized complication of tuberculosis (TB) meningitis, typically associated with spinal arachnoiditis and disruption of cerebrospinal fluid dynamics. Although the exact mechanisms are not fully understood, early onset during the acute phase of illness is uncommon.

We report the case of a 30-year-old male who presented with progressive neurological symptoms one week after initial evaluation for headache and fever following travel to a TB-endemic region. He developed gait unsteadiness, tremors, and nystagmus and was subsequently found to have a contrast-enhancing lesion near the cerebral aqueduct with early hydrocephalus. Empirical anti-tuberculous therapy and corticosteroids were commenced, but the patient deteriorated, requiring intensive care admission, intubation, and tracheostomy. Over the course of his admission, he developed quadriplegia and was diagnosed with syringomyelia on MRI, involving cervical cord segments C1-C4, alongside extensive spinal cord swelling.

Management included modified anti-tuberculous therapy in the context of drug-induced liver injury, ventricular drainage, and immunomodulatory therapy with infliximab for suspected paradoxical TB response. Neurosurgical input advised conservative management of the syrinx due to its small, loculated nature. The patient remained under multidisciplinary care, including complex ventilation support and neurorehabilitation. Decisions regarding surgical versus non-surgical management are guided by factors such as the syrinx’s size, location, underlying cause, and the patient’s clinical status. In general, surgical intervention is reserved for those with progressive symptoms or worsening radiological findings.

## Introduction

Tuberculosis (TB) continues to represent a significant global health challenge and remains one of the leading infectious causes of death worldwide. It is estimated that approximately one-quarter of the global population is infected with *Mycobacterium tuberculosis*. In 2023, an estimated 10.8 million individuals developed active disease, comprising 6.0 million men, 3.6 million women, and 1.3 million children [1]. When TB involves the central nervous system (CNS), it manifests as TB meningitis (TBM), the most severe form of extrapulmonary TB, which carries high rates of morbidity and mortality. *Mycobacterium tuberculosis* gains access to the CNS by crossing protective barriers, triggering a pronounced immune-mediated inflammatory response involving both local and systemic pathways [2].

TBM accounts for approximately 1% of all extrapulmonary TB cases. In regions with low TB prevalence, such as high-income countries, TBM is responsible for around 6% of all meningitis cases. Conversely, in areas with high TB endemicity, TBM contributes to nearly one-third to half of all bacterial meningitis presentations [3].

Syringomyelia is a rare but recognized complication of TBM, occurring in approximately 1-2% of active TB cases [4]. It is most commonly secondary to spinal arachnoiditis (arachnoid membrane inflammation). This chronic inflammatory process develops following meningitis, which can disrupt cerebrospinal fluid (CSF) circulation at the foramen magnum or along the spinal cord, leading to syrinx formation. Although CSF flow obstruction is considered central to its pathogenesis, the exact mechanisms remain incompletely understood [5].

Timely use of corticosteroids such as dexamethasone has been shown to improve survival in TBM by limiting inflammation. While anti-tuberculous treatment may reduce the inflammatory process and occasionally prevent syrinx development, its effectiveness is generally confined to the active phase of infection. Surgical options may be required for selected patients, especially those with symptomatic or progressive syringomyelia. These include shunting techniques (e.g., syringo-peritoneal or syringo-pleural shunts) and decompressive procedures such as adhesiolysis or duraplasty [4].

This report discusses a case of a 30-year-old male who developed syringomyelia shortly after the onset of TBM, highlighting the clinical approach and multidisciplinary considerations in managing this rare early complication.

This case was presented as a poster at the Association of British Neurologists conference in May 2025 and will be showcased in their monthly conference editorial.

## Case presentation

A 30-year-old male returned to the United Kingdom following a one-month stay in Nigeria, during which he was treated for malaria with artemether-lumefantrine. He presented shortly after with a two-week history of fever and headache in May 2024. His initial bloods were unremarkable (Table 1). Malaria screens (three sets) were negative, and he was discharged with safety netting and a follow-up plan in the clinic.

**Table 1:** Lab parameters on initial review WCC: white cell count, Hb: hemoglobin, Na: sodium, K: potassium, eGFR: estimated glomerular filtration rate

Test	Result	Reference range
WCC	9.8	4-10
Hb	134 g/L	115-150 g/L
Neutrophil count	7.78	2-7.5
Platelets	461	154-400
Na	133 mmol/L	135-145 mmol/L
K	4 mmol/L	3.5-5 mmol/L
Urea	4 mmol/L	2.5-7.8 mmol/L
eGFR	>90	>90
C-reactive protein	1 mg/L	<5 mg/L
Malaria screening test	Negative	Negative
Blood culture	Negative growth after 48 hours of incubation	No growth

**Figure 1:**
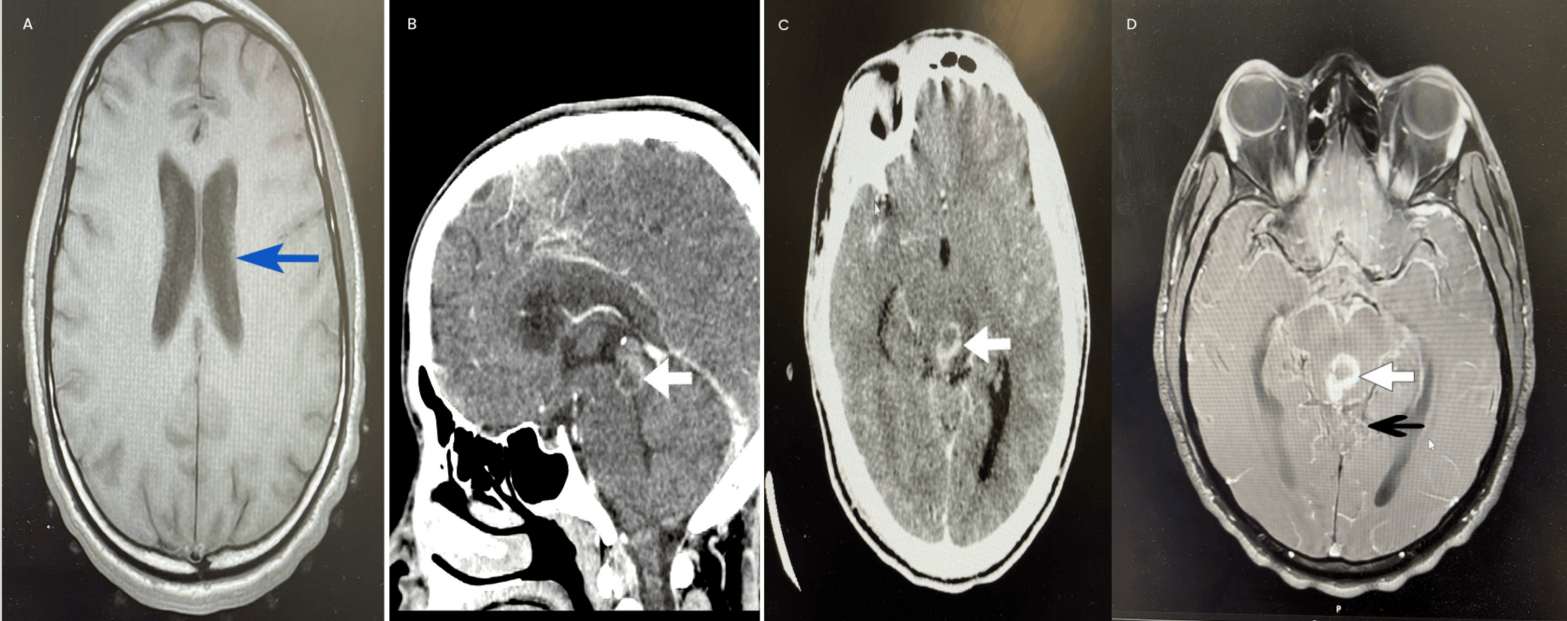
(A) Axial CT head showing dilatation of the lateral ventricles, consistent with early hydrocephalus. (B) Sagittal contrast-enhanced CT demonstrating a 17 × 16 mm peripherally enhancing lesion centered on the cerebral aqueduct, with a central non-enhancing component. (C) Axial CT at the level of the midbrain showing the same lesion in cross-section. (D) Post-contrast T1-weighted axial MRI demonstrating intense enhancement of the circular periaqueductal lesion, with subtle leptomeningeal enhancement within the quadrigeminal and prepontine cisterns. CT: computed tomography, MRI: magnetic resonance imaging, blue arrow: dilated lateral ventricle, white arrow: solitary peripherally enhancing mass lesion, black arrow: leptomeningeal enhancement

**Figure 2:**
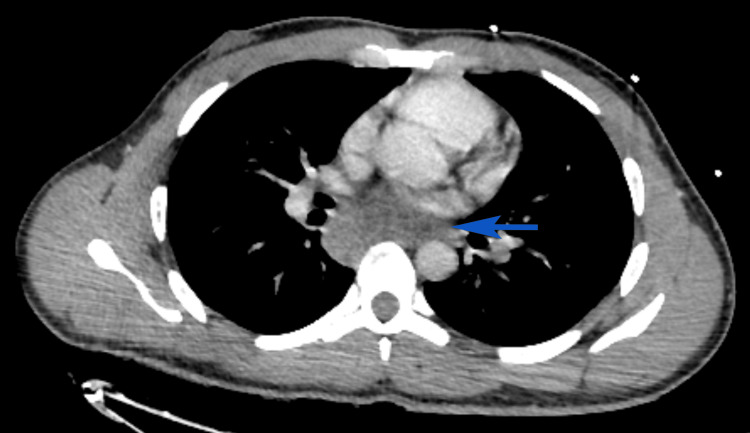
CT thorax axial view demonstrating a heterogeneous, septated posterior mediastinal mass measuring 31 × 66 mm in axial dimensions and 61 mm in craniocaudal length. CT: computed tomography, blue solid arrow: heterogeneous posterior mediastinal mass

He re-presented approximately one week after the initial evaluation in the clinic with worsening symptoms, including neck stiffness, persistent nausea and vomiting, and evolving neurological signs. He reported an unsteady gait, tremors, and increasing lethargy. Upon examination, he exhibited horizontal nystagmus and past-pointing. A CT brain revealed a peripherally enhancing lesion with central hypodensity near the cerebral aqueduct, involving the anterior commissure and both superior and inferior colliculi, with features of early hydrocephalus (Figure 1). He also underwent an MRI of the brain after the initial CT head, later the same day, which showed the same peripherally enhancing lesion with central hypodensity and leptomeningeal enhancement (Figure 2).

**Figure 3:**
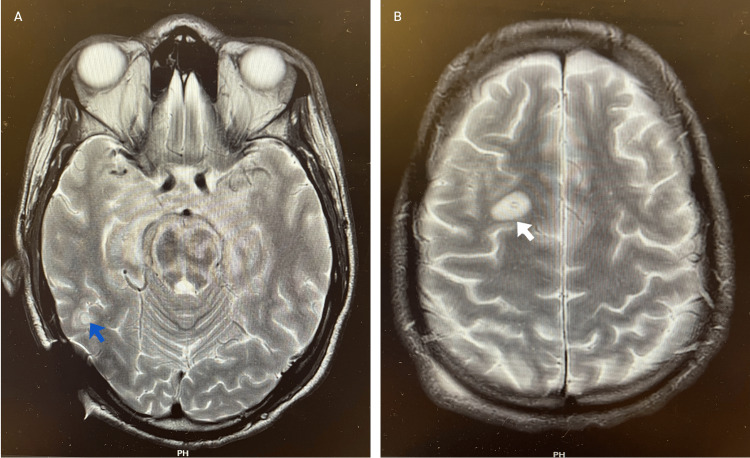
MRI head T2-weighted axial images demonstrating new lesions: (A) round hyperintense with a slight hypointense rim in the left posterior cerebrum and left-side external ventricular drain site. (B) rounded hyperintense lesion in the left frontal white matter. MRI: magnetic resonance imaging, blue arrow: lesion in the left posterior cerebrum, white arrow: lesion in the left frontal white matter

He was referred urgently to a tertiary neurosurgical center and admitted to the intensive care unit (ICU) the same day. A ventricular drain was inserted for hydrocephalus management immediately after transfer on day 1 of the ICU stay. Empirical anti-tuberculous therapy (HRZE: isoniazid, rifampicin, pyrazinamide, and ethambutol) and intravenous dexamethasone (0.4 mg/kg/day) were commenced, guided by Thwaites’ score and CT TAP findings, which demonstrated a posterior mediastinal septated mass (Figure 3). Empirical antimicrobial cover included ceftriaxone, metronidazole, and vancomycin. TB polymerase chain reaction (PCR) from CSF was negative. In addition, CSF results showed normal protein and glucose and two to three lymphocytes. Microbiological confirmation of TB was obtained two months into the patient’s ICU admission, with a positive *Mycobacterium tuberculosis* PCR from the biopsy performed via ultrasound-guided transoesophageal approach, targeting the posterior mediastinal retrocardiac lymph node mass (Figure 2).

During his ICU stay, the patient deteriorated, with a decline in Glasgow Coma Scale (GCS) to 8/15 and development of quadriparesis, with Medical Research Council (MRC) grade 3 within the first week. He was intubated, ventilated, and subsequently underwent tracheostomy. Liver function deteriorated, with a peak alanine transaminase of 455 U/L, consistent with a drug-induced liver injury. Anti-tuberculous therapy was adjusted to a liver-sparing regimen comprising linezolid, levofloxacin, ethambutol, and clofazimine with multidisciplinary team input. Clofazimine was temporarily withheld due to enteral access issues, during which time meropenem and co-amoxiclav were administered. A ventriculoperitoneal shunt was inserted following dislodgement of the external ventricular drain. Standard therapy with HRZE was resumed after normalization of liver function two months later.

By June 2024, the patient showed further neurological decline with a quadriparesis of MRC grade 2, raising concern for a paradoxical tuberculous reaction. MRI demonstrated new nodular intracranial lesions, worsening brainstem edema, and extensive leptomeningeal enhancement (Figure 3). One dose of infliximab (5 mg/kg) was administered following multidisciplinary consensus.

**Figure 4:**
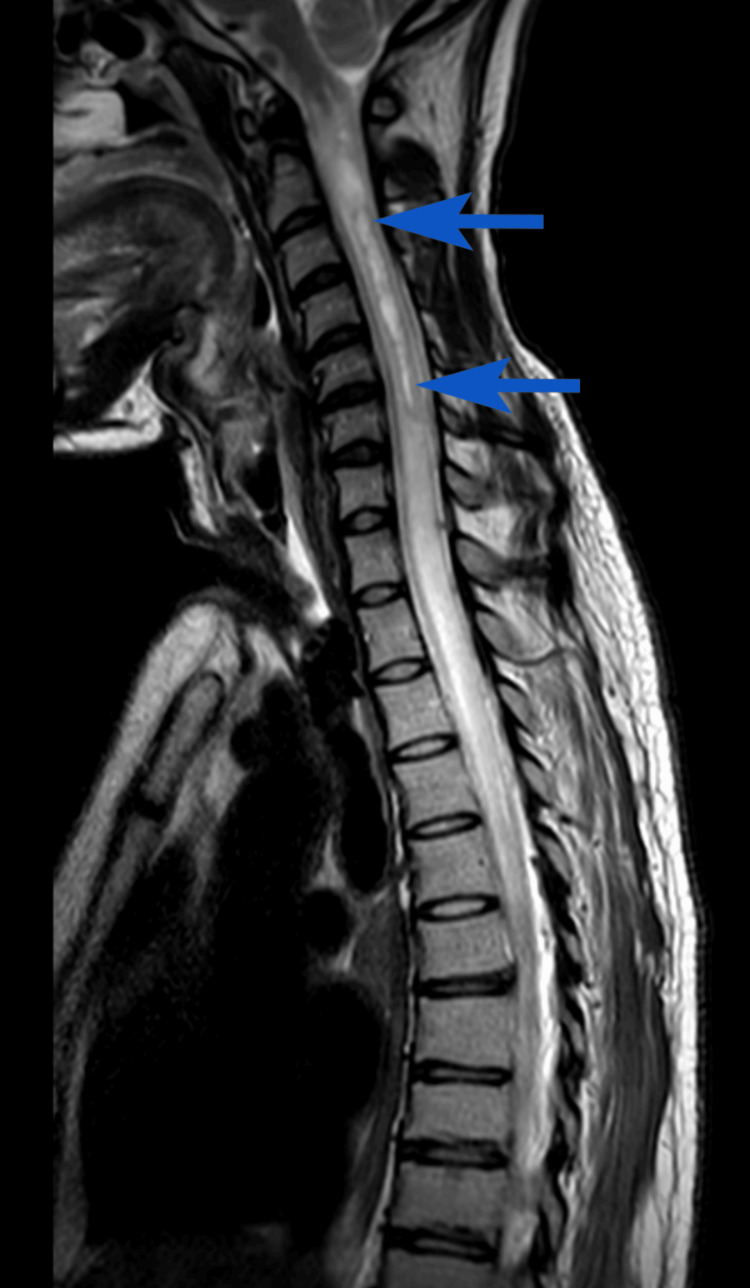
MRI spine sagittal T2-weighted view demonstrating a syrinx extending from C1 to C4. MRI: magnetic resonance imaging, blue arrow: syrinx

He remained in the ICU with a tracheostomy placed for long-term ventilation. Over the course of the next two months in the ICU, his quadriplegia progressed (MRC grade 0). An MRI of the spine revealed progressive spinal cord swelling with the development of a cervical syrinx at C1-C4 (Figure 4). These changes were attributed to a combination of inflammatory injury, impaired CSF dynamics, and fibrosis. After further multidisciplinary team discussion, he received two further doses of infliximab (5 mg/kg). The patient was discussed with the spinal surgical team, who reviewed the imaging and advised conservative management given the small, loculated nature of the syrinx.

**Figure 5:**
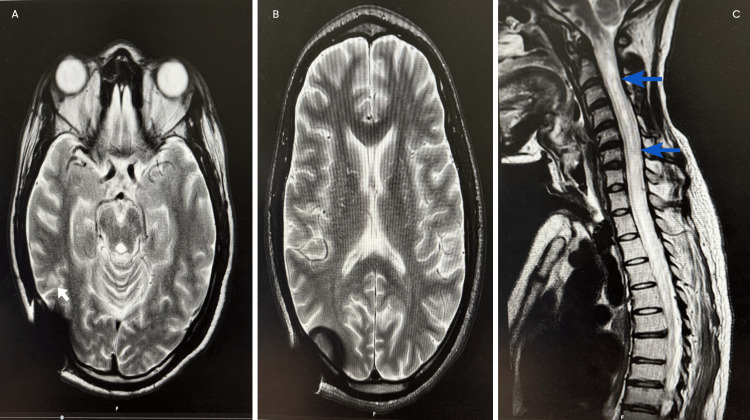
(A) Axial T2-weighted MRI demonstrating reduction in the size of a round hyperintense lesion in the left posterior cerebrum. (B) Axial T2-weighted MRI showing complete resolution of the left frontal white matter lesion. (C) Sagittal T2-weighted MRI of the spine showing a stable syrinx extending from C1 to C4. MRI: magnetic resonance imaging, white arrow: lesion in the left posterior cerebrum, blue arrow: syrinx

Subsequent imaging after about 1.5 months in October 2024 showed improvement in intracranial lesions and non-progression of the syrinx (Figure 5).

The patient’s consciousness improved (GCS-10/15), though there was minimal recovery of limb function (MRC grade 1) by October 2024. Anti-tuberculous treatment was continued for a total period of 12 months under infectious disease team supervision. He was referred to the complex ventilation team for tracheostomy weaning and to specialist neurorehabilitation services for long-term care.

## Discussion

Syringomyelia is a relatively rare complication of TBM, typically occurring late in the disease course. The reported interval between TBM and syrinx formation ranges from 7 to 28 years [6]. In this case, however, the patient developed a syrinx extending from C1 to C4 within six months of symptom onset. The clinical progression was also notably rapid following the initial presentation. Early empirical management with anti-tuberculous therapy and corticosteroids was initiated as per the infectious diseases team’s recommendation. Early initiation of such therapy has been shown to improve survival outcomes in TBM by controlling spinal inflammation [4].

In addition to medical management, various surgical options have been described for syringomyelia, including syrinx drainage via shunting procedures (syringo-peritoneal, syringo-pleural, or syringo-subarachnoid) and decompressive interventions such as arachnoid adhesiolysis, subpial suction, or duraplasty [4]. Treatment aims to address the underlying pathophysiology, restore normal CSF flow, and improve CSF dynamics. The decision between operative and non-operative management depends on the syrinx’s location, size, etiology, and symptomatology. Surgical intervention is generally indicated in symptomatic patients with worsening neurological function or progressive radiological changes [7]. In our case, following spinal surgical review, a conservative approach was adopted due to the small and loculated nature of the syrinx. Subsequent follow-up imaging demonstrated no further progression, supporting the decision to avoid surgical intervention.

Tumor necrosis factor alpha (TNF-α) is a key cytokine involved in granuloma formation, a protective immunological mechanism against Mycobacterium tuberculosis that restricts bacterial proliferation. Paradoxically, TNF-α can also drive excessive inflammation and tissue injury. This immune response may lead to clinical or radiological deterioration after initial improvement with treatment, a condition termed PR. PR is relatively common, with radiological worsening reported in up to 90% of TBM cases within two months of therapy initiation.

The use of TNF-α inhibitor monoclonal antibodies to manage severe, corticosteroid-resistant PR in adults with CNS TB was first described in 2008, resulting in a favorable outcome for that patient. Since then, most reported cases have involved PR unresponsive to corticosteroids, with almost all demonstrating clinical improvement following treatment with infliximab or adalimumab. Improvement after infliximab initiation is often gradual and may be preceded by radiological resolution. Despite optimal anti-tuberculous and corticosteroid therapy, morbidity in CNS TB remains high due to persistent inflammation [8]. Corticosteroids remain the mainstay of PR management; however, in severe steroid-refractory cases, infliximab is beneficial [9].

In this patient, despite adequate anti-tuberculous therapy and corticosteroids, there was progressive clinical and radiological deterioration. The intracranial tuberculomas increased in both size and number (Figure 3), and the patient’s quadriparesis worsened from MRC grade 3 to grade 2 within the first two months of treatment. These findings were consistent with a PR or immune reconstitution inflammatory syndrome. The patient subsequently received three doses of infliximab (5 mg/kg) over a period of three months, alongside continued corticosteroid therapy, following multidisciplinary consensus. By the end of the infliximab course, the cerebral lesions had reduced in size and number (Figure 5), the syrinx remained stable, and the patient demonstrated neurological improvement, with recovery of consciousness and limb strength (MRC grade 1).

## Conclusions

This case demonstrates that syringomyelia can develop unusually early following tuberculous meningitis. Conservative management was appropriate given the small, loculated syrinx, which remained stable on follow-up imaging. Furthermore, infliximab may be considered in severe paradoxical reactions unresponsive to corticosteroids. Early imaging, timely medical therapy, and multidisciplinary collaboration are crucial, while surgical intervention should be reserved for progressive cases.
